# Chlorine-Rich Na_6−*x*_PS_5−*x*_Cl_1+*x*_: A Promising Sodium Solid Electrolyte for All-Solid-State Sodium Batteries

**DOI:** 10.3390/ma17091980

**Published:** 2024-04-24

**Authors:** Yi Zhang, Haoran Zheng, Jiale You, Hongyang Zhao, Abdul Jabbar Khan, Ling Gao, Guowei Zhao

**Affiliations:** 1 College of Chemistry and Chemical Engineering, Huanggang Normal University, Huanggang 438000, China; legend10086@163.com (Y.Z.);; 2 School of Chemistry, Xi’an Jiaotong University, Xi’an 710049, China

**Keywords:** Na_6_PS_5_Cl, sodium solid electrolyte, all-solid-state batteries, ionic conductivity, high electrochemical stability

## Abstract

Developing argyrodite-type, chlorine-rich, sodium-ion, solid-state electrolytes with high conductivity is a long-term challenge that is crucial for the advancement of all-solid-state batteries (ASSBs). In this study, chlorine-rich, argyrodite-type Na_6−*x*_PS_5−*x*_Cl_1+*x*_ solid solutions were successfully developed with a solid solution formation range of 0 ≤ *x* ≤ 0.5. Na_5.5_PS_4.5_Cl_1.5_ (*x* = 0.5), displaying a highest ionic conductivity of 1.2 × 10^−3^ S/cm at 25 °C, which is more than a hundred times higher than that of Na_6_PS_5_Cl. Cyclic voltammetry and electrochemical impedance spectroscopy results demonstrated that the rich chlorine significantly enhanced the ionic conductivity and electrochemical stability, in addition to causing a reduction in activation energy. The Na_5.5_PS_4.5_Cl_1.5_ composite also showed the characteristics of a pure ionic conductor without electronic conductivity. Finally, the viability of Na_5.5_PS_4.5_Cl_1.5_ as a sodium electrolyte for all-solid-state sodium batteries was checked in a lab-scale ASSB, showing stable battery performance. This study not only demonstrates new composites of sodium-ionic, solid-state electrolytes with relatively high conductivity but also provides an anion-modulation strategy to enhance the ionic conductivity of argyrodite-type sodium solid-state ionic conductors.

## 1. Introduction

Sodium-ion batteries (SIBs) have been considered a highly promising alternative to commercialized lithium-ion batteries (LIBs) due to the natural abundance of sodium resources (2.64 wt% for Na against 0.0017 wt% for Li) and their higher safety levels with similar charge/discharge mechanism [1]. Unfortunately, conventional SIBs face environmental and safety issues originating from the effumability and leakage of flammable organic liquid electrolytes [2,3]. Employing solid-state electrolytes (SSEs) instead of liquid organic electrolytes is a viable solution to the battery issues, since inorganic SSEs are non-volatile and non-flammable [4,5,6]. Therefore, the rational design of advanced sodium SSEs has been treated as a reliable way to achieve high-performance SIBs. 

Sodium-based SSEs have been well developed in recent year and can be roughly divided into oxide-type and sulfide-type SSEs. Oxide-type SSEs such as *β*-alumina [7,8], NASICON–type Na_1+*x*_Zr_2_Si*_x_*P_3−_*_x_*O_12_ [9,10], and Na_2_*M*_2_TeO_6_ (*M* = Zn, or Mg) [11,12,13] exhibit good ionic conductivity of 10^−4^–10^−3^ S/cm. However, their poor interfacial compatibilities with electrodes lead to high contact resistances, which hinder the practical fabrication of all-solid-state sodium batteries at room temperature [14]. In contrast, sulfide materials are highly mechanically deformable and have excellent interfacial contact with active materials simply upon cold pressing [15,16]. The development of sodium-based sulfide conductors can be dates to 1992, when a homolog of a thio-LISICON-type, sulfide-based Na_3_PS_4_ sodium-ionic conductor with relatively high ionic conductivity was reported not long after the discovery of the well-known Li_3_PS_4_ lithium-ionic conductor in 1984 [17,18]. Since then, numerous studies have focused on the investigation of sulfide-based SSEs for SIBs [19,20,21]. Analogous ionic conductors such as Na_3_SbS_4_ have been synthesized and described in detail [22], while element-doping and defect-introducing strategies have been utilized to promote their electrochemical performance and air stability [2,15,23,24,25,26,27,28]. Moreover, inspired by the remarkable super-high ionic conductivity of thio-phosphate-type Li_10_GeP_2_S_12_ (LGPS, 12 mS/cm) sulfide electrolytes [29,30,31,32], a series of Na_10_*M*P_2_S_12_ (*M* = Ge, Si, Sn) sulfide compounds was predicted through first-principles calculations, and new Na-ion conductors, Na_10+*x*_Sn_1+*x*_P_2−_*_x_*S_12_ (*x* = 0, 1) with tetragonal phases, have been successfully synthesized, possessing high ionic conductivity at ambient temperature [33,34,35,36]. However, Sn^4+^ in the SSE is potentially active when equipped with a highly reductive sodium metal anode, which might result in interfacial instability [37]. Therefore, a highly stable sodium SSE with high ionic conductivity is urgently needed. 

Argyrodite-type conductors with high ionic conductivity and stable interfacial properties have been considered as promising electrolytes for all-solid-state batteries due to the absence of transition-metal species [38,39]. The Li_6_PS_5_*X* lithium-based conductor (*X* = Cl, Br, and I) showed their promising properties as an SSE, whereas few reports are available on its sodium analogs. Nevertheless, in previous reports, an argyrodite-type conductor, Na_6_PS_5_Cl, was synthesized [40]. Unexpectedly, the ion conductivity of Na_6_PS_5_Cl was only 1.2 × 10^−5^ S/cm, which was significantly lower than that of its Li_6_PS_5_Cl analog (3 × 10^−3^ S/cm) [41]. The electrochemical window of Na_6_PS_5_Cl is only 1.3 V, which makes it impossible for practical use. Increasing the degree of structure disorder by raising the halide content of electrolytes can strengthen the diffusivity of ions in the SSE [41,42]. Therefore, increasing the Cl and Na vacancy contents in Na_6_PS_5_Cl is favorable for the promotion of Cl^−^/S^2−^ disordering, as well as Na-ion mobility.

In this work, a highly conductive chlorine-rich sodium solid electrolyte, Na_5.5_PS_4.5_Cl_1.5_, was successfully synthesized by increasing Cl and Na vacancy contents with an outstanding conductivity of 1.2 × 10^−3^ S/cm at room temperature, which is almost ten-fold greater than that of pristine Na_6_PS_5_Cl. The introduction of Cl and Na vacancy contents in Na_6_PS_5_Cl induced a significant disordering of S^2−^/Cl^−^ arrangement, leading to the lowering of the activation barriers. Moreover, the electrochemical window of the Cl-rich SSE increased to 2 V, which makes the SSE a practical candidate for ASSBs. We demonstrated the availability of the chlorine-rich Na_5.5_PS_4.5_Cl_1.5_ SSE by assembling a Na_3_V_2_(PO_4_)_3_//Na_5.5_PS_4.5_Cl_1.5_//Na full battery, indicating the practical application value of the new Na_5.5_PS_4.5_Cl_1.5_ sulfide electrolyte for ASSBs.

## 2. Materials and Methods

### 2.1. Materials Synthesis

Na_6−_*_x_*PS_5−_*_x_*Cl_1+_*_x_* (*x* = 0, 0.1, 0.2, 0.3, 0.4, 0.5, and 0.6) was synthesized by a high-temperature, solid-state reaction method. Stoichiometric Na_2_S, P_2_S_5_, and NaCl (all chemicals with a purity of 99.99%; Macklin Biochemical Co., Ltd., Shanghai, China) were weighed by chemical stoichiometry in an Ar-filled glovebox (<1 ppm of O_2_, H_2_O) and sealed in a 45 mL ZrO_2_ pot with 15 ZrO_2_ balls with 10 mm diameters and 40 ZrO_2_ balls with 5 mm diameters. The raw materials were ball-milled with a planetary ball mill apparatus (YXQM-0.45L, Changsha Mitrcn Instrument Equipment Co., Ltd., Changsha, China) at a rotational speed of 400 rpm for 8 h. Then, the obtained precursors were pelleted at 20–30 MPa in an Ar-filled glovebox (<1 ppm of O_2_, H_2_O), sealed in an evacuated Pyrex tube at pressures below 2 × 10^−3^ Pa, and sintered at 450 °C for 12 h with a heating rate of 2 °C/min. After naturally cooling to room temperature, the as-synthesized Na_6−_*_x_*PS_5−_*_x_*Cl_1+_*_x_* was transferred to a glovebox and mixed by hand with mortar and pestle for 15 min for further characterization and battery assembly.

### 2.2. Methods

Powder XRD measurements were conducted using a diffractometer equipped with CuKα_1_ radiation (XRD-6010, Shimadzu, Tokyo, Japan) to ascertain the phase compositions of the synthesized powders. Prior to XRD measurements, samples were prepared in a glove box and sealed with polyimide film (Dongguan Meixin Co., Ltd., Dongguan, China) to shield them from moisture during the measurements. The diffraction data were collected in the 2θ range of 15° to 50° with step widths of 0.01°. The morphology and elemental mapping analyses for the composite powders were obtained using energy-dispersive X-ray spectroscopy (EDS, Bruker QUANTAX, Berlin, Germany), and the obtained samples were examined using a scanning electron microscope (SEM) (JEOL Ltd., JSM-6390, Tokyo, Japan).

The ionic conductivities of the cylindrical solid electrolyte (SE) pellets were assessed using the AC impedance technique with a cell (stainless steel/SE/stainless steel), employing pressurizable sealing molds and measured between 25 and 85 °C. This measurement was repeated two or three times, applying 15 mV in the frequency range of 7 MHz to 1 Hz and utilizing a potentiostat electrochemical interface (Bio-Logic SAS, VSP-300, Claix, France). SE pellets with a diameter of 10 mm were fabricated under a pressure of 303 MPa using a polyaryletherketone mold. Electrochemical window measurements were conducted using sodium/solid electrolyte/sodium cells. Typically, 100 mg of SE was pressed at 150 MPa to form a solid pellet. Na metal foil (purity: 99.9%, thickness: 0.1 mm, diameter: 5 mm; 99.9%, MTI Corporation, Shenzhen, China) was placed on the top side of the electrolyte pellet and further pressed at 150 MPa. The electrochemical stability of Na_6−_*_x_*PS_5−_*_x_*Cl_1+*x*_ series was determined over a wider potential window, and a cyclic voltammetry (CV) test was conducted at a scan rate of 0.1 mV/s between −2.5 and 2.5 V for comparison of the voltage stability region. The stack pressure applied during the tests was about 5 MPa.

An all-solid-state battery employing the obtained chlorine-rich sodium-ionic conductor with the highest ionic conduction as the electrolyte was assembled under a pressure of 6 MPa within an Ar-filled glovebox to evaluate its charge–discharge performance. The battery assembly method and the utilized cathode materials were akin to those documented in previous reports [43]. The details are as follows: Na metal foil served as the anode, while a composite mixture comprising Na_3_V_2_(PO_4_)_3_ (NVP) and Na_5.5_PS_4.5_Cl_1.5_ (NPSC-6) (NVP:NPSC-6 = 7:3) was employed as the cathode. Prior to use, a sieve with a 10 μm mesh was utilized to eliminate large-particle-size powder in Na_5.5_PS_4.5_Cl_1.5_. A pellet with a diameter of 10 mm was produced by cold pressing approximately 100 mg of Na_5.5_PS_4.5_Cl_1.5_ powder. Subsequently, Na foil (purity: 99.9%; thickness: 0.1 mm; diameter: 5 mm; 99.9%; MTI Corporation, Shenzhen, China) with a Cu mesh (purity: >99.9%; thickness: 0.045 mm; diameter: 8 mm; pore size: 0.4 mm × 1.5 mm; MTI Corporation, Hefei, China) was successively pressed onto one side of the pellet. On the opposite side of the pellet, the cathode composite (8 mg) with Al mesh (purity > 99.9%; thickness: 0.055 mm; diameter: 10 mm; pore size; 0.4 mm × 1.5 mm; MTI Corporation, Hefei, China) and an Al foil current collector (thickness: 0.1115 mm; diameter: 10 mm; purity: >99.9%, MTI Corporation, Hefei, China) were then pressed. Charge–discharge measurements were conducted at 25 °C, between 1.4 and 1.8 V, and with a current density of 0.03 mA cm^−2^ (0.05 C) using the LANHE CT2001A charge–discharge system (Wuhan LAND Electronics Co., Wuhan, China). The stack pressure applied during the tests was about 5 MPa.

## 3. Results and Discussion

### 3.1. Physical Characterization of Na_6−x_PS_5−x_Cl_1+x_

**Figure 1:**
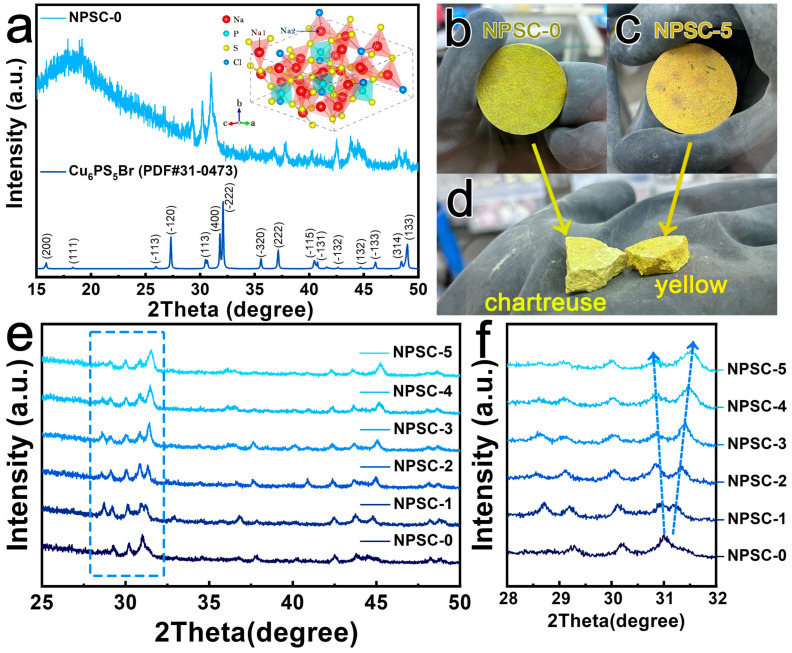
(**a**) XRD patterns of the Na_6_PS_5_Cl sample and argyrodite-Cu_6_PS_5_Br (inset represents the crystal structure model of Cu_6_PS_5_Br). (**b**,**c**) Digital images of the synthesized Na_6−*x*_PS_5−*x*_Cl_1+*x*_ (*x* = 0 and 0.5) solid block. (**d**) Comparison of inner color of Na_6−*x*_PS_5−*x*_Cl_1+*x*_ (*x* = 0 and 0.6 samples. (**e**) XRD patterns of Na_6−*x*_PS_5−*x*_Cl_1+*x*_ (*x* = 0, 0.1, 0.2, 0.3, 0.4, and 0.5, denoted as NPSC-0, NPSC-1, NPSC-2, NPSC-3, NPSC-4, and NPSC-5, respectively). (**f**) Enlarged XRD patterns in the 2*θ* range of 28–32° from the dotted boxes in (**e**), the arrows clearly show regular shifting in one direction with increasing Cl content, indicating the formation of solid solutions.

Na_6−_*_x_*PS_5−_*_x_*Cl_1+*x*_ (*x* = 0, 0.1, 0.2, 0.3, 0.4, 0.5, or 0.6, denoted as NPSC-0, NPSC-1, NPSC-2, NPSC-3, NPSC-4, NPSC-5, and NPSC-6, respectively) samples were prepared by a solid-state reaction. The XRD patterns of the NPSC-0 sample were similar to those of the related argyrodite-type Cu_6_PS_5_Br, which displayed a monoclinic phase with a Cc space group [44,45]. A structural model of atomic Na_6_PS_5_Cl was simulated by replacement of Cu and Br in Cu_6_PS_5_Br with Na and Cl through structural refinement using Vesta software (version 3) [46], as displayed in the inset of Figure 1a. Two different Na^+^ sites were tetrahedrally coordinated. The Na1 site was surrounded by four S atoms, while the Na2 site was encircled by three S atoms and one Cl atom. The obtained NPSC-0 product was a solid block with chartreuse color (Figure 1b), while the outer and inner layers turned yellow in the NPSC-5 sample (Figure 1c,d). Comparisons of XRD patterns with those of other related compounds (such as tetragonal Na_3_PS_4_, cubic Na_3_PS_4_, and NaCl) are displayed in Figure S1, proving that no Na_3_PS_4_ or NaCl impurities were included in the NPSC-0 sample. With the increase in *x* in Na_6−_*_x_*PS_5−_*_x_*Cl_1+*x*_, the (400) peak gradually shifted to lower 2*θ* angles, while the ($\overset{-}{2}$22) peak migrated to higher 2*θ* angles, indicating the formation of solid solutions in Na_6−_*_x_*PS_5−_*_x_*Cl_1+*x*_. Further introduction of additional chlorine into the Na_6−_*_x_*PS_5−_*_x_*Cl_1+*x*_ structure caused exsolvation of NaCl impurity at *x* = 0.6 (Figure S2), resulting a decrease in ionic conductivity. Thus, under the present experimental synthesis conditions, it was shown that Na_5.5_PS_4.5_Cl_1.5_ (*x* = 0.5) is the limit of solid solution formation in the Na_6−_*_x_*PS_5−_*_x_*Cl_1+*x*_ system. In other words, Na_6−_*_x_*PS_5−_*_x_*Cl_1+*x*_ formed solid solutions in the range of 0 ≤ *x* ≤ 0.5.

**Figure 2:**
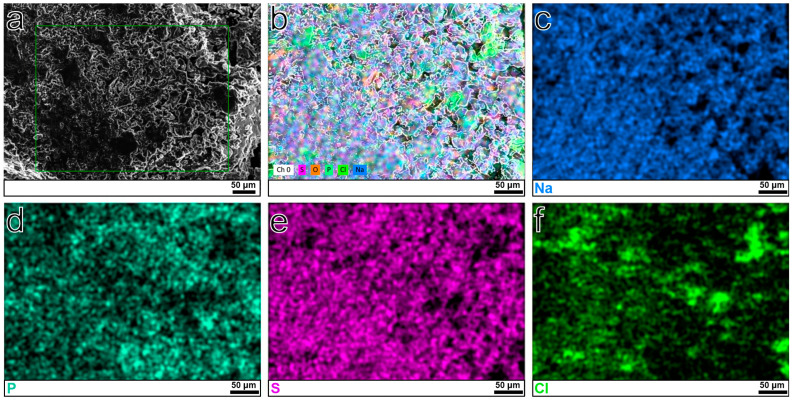
(**a**) SEM image of NPSC-5 sample. (**b**–**f**) EDS element mapping of Na, P, S, and Cl in NPSC-5 sample.

The morphology analysis results showed that the particle sizes of the NPSC-0, NPSC-1, NPSC-2, NPSC-3, NPSC-4 and NPSC-5 samples were in the range of 5 to 50 μm, which was gradually reduced with the increase in the Cl^−^/S^2−^ ratio, as shown in Figure 2a and Figure S3. The elemental mapping analyses shown in Figure 2b–f indicate the presence and uniform distribution of Na, P, S, and Cl elements within the NPSC-5 sample.

### 3.2. Electrochemical Performance of Na_6−x_PS_5−x_Cl_1+x_ Electrolyte

**Figure 3:**
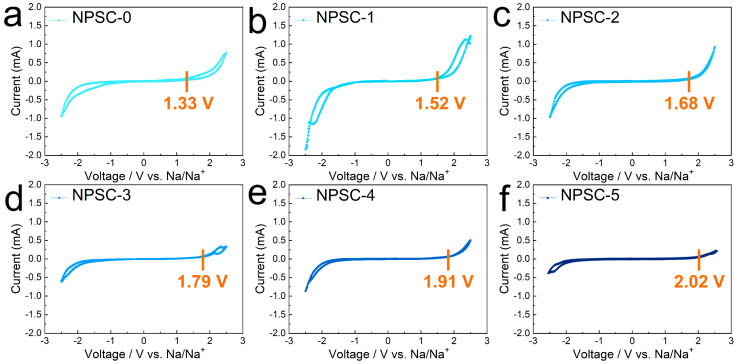
CV curves of (**a**) Na/NPSC-0/Na, (**b**) Na/NPSC-1/Na, (**c**) Na/NPSC-2/Na, (**d**) Na/NPSC-3/Na. (**e**) Na/NPSC-4/Na, and (**f**) Na/NPSC-5/Na with Na_6−*x*_PS_5−*x*_Cl_1+*x*_ as analytical materials (*x* = 0, 0.1, 0.2, 0.3, 0.4, and 0.5, denoted as NPSC-0, NPSC-1, NPSC-2, NPSC-3, NPSC-4, and NPSC-5, respectively) and a scan rate of 0.1 mV/s.

The electrochemical stability of Na_6−_*_x_*PS_5−_*_x_*Cl_1+*x*_ was determined for comparison of the voltage stability region, the results of which are shown in Figure 3. The NPSC-0 sample showed a small stable region only at 1.33 V (Figure 3a). With the increase in *x* in Na_6−*x*_PS_5−*x*_Cl_1+*x*_, the voltage stability region widened gradually. When *x* = 0.1, the stable region was at 1.52 V; when *x* = 0.2, the stable region shifted to 1.68 V. It kept increasing to 1.79 and 1.91 V in the NPSC-3 and NPSC-4 samples, respectively. The NPSC-5 sample exhibited a maximum stable voltage of 2.02 V, indicating that promoting the Cl content in Na_6−_*_x_*PS_5−_*_x_*Cl_1+*x*_ favors the widening of the operating potential window.

**Figure 4:**
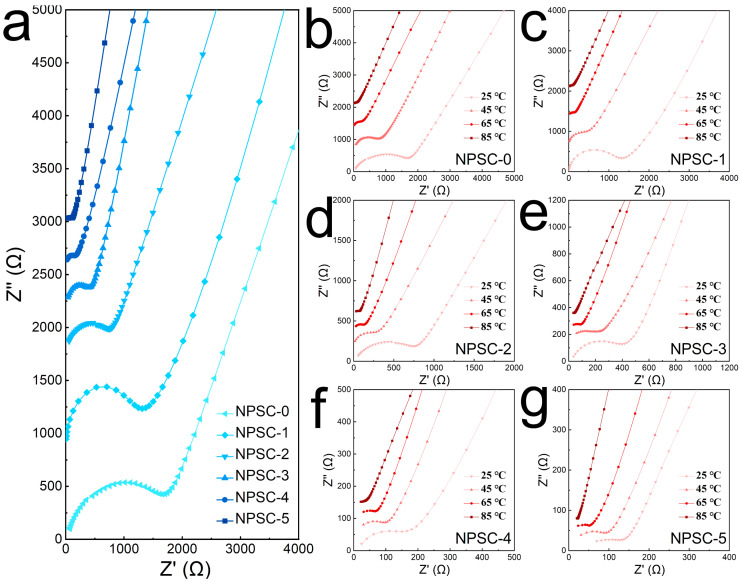
(**a**) Impedance plots of Na_6−*x*_PS_5−*x*_Cl_1+*x*_ samples (*x* = 0, 0.1, 0.2, 0.3, 0.4, and 0.5, denoted as NPSC-0, NPSC-1, NPSC-2, NPSC-3, NPSC-4, and NPSC-5, respectively) obtained at 25 °C. (**b**) Impedance plots of the complex impedance responses of (**b**) NPSC-0, (**c**) NPSC-1, (**d**) NPSC-2, (**e**) NPSC-3, (**f**) NPSC-4, and (**g**) NPSC-5 samples measured at 25, 45, 65, and 85 °C, respectively.

Figure 4 illustrates the complex impedance spectra of the uniaxially cold, isostatic pressed powder samples obtained in the study. The impedance plots exhibit a semicircle pattern accompanied by a spike observed at high and low frequencies, respectively [43]. These features correspond to the total resistance (combining bulk and grain boundary contributions) and electrode resistances [47]. The semicircle represents ionic transport within the materials, while the spike is indicative of ion-blocking behavior at the interfaces between the electrodes and electrolyte. The equivalent circuits used for impedance analysis, as depicted in Figure S4, were derived from the ZView program [47]. Additionally, the semicircles observed at higher frequencies, displaying capacitances of 10^−10^ F, suggest total resistance, with a combined contribution from both bulk and grain boundaries [48].

The ionic conductivity of Na_6−_*_x_*PS_5−_*_x_*Cl_1+*x*_ as analytical material was characterized using the AC impedance method. All the impedance spectra contained formed semicircle pattern at high frequencies attributed to the charge-transfer resistance and an inclined line at low frequencies associated with diffusion processes in the electrodes. With the increase in *x* in Na_6−_*_x_*PS_5−_*_x_*Cl_1+*x*_ (0 ≤ *x* ≤ 0.5), the semicircle diminished gradually, leading to a reduction in resistances by introducing rich Cl contents and facilitating Na^+^ diffusion in Na_6−_*_x_*PS_5−_*_x_*Cl_1+*x*_ (Figure 4a). The calculated ionic conductivities rose gradually with increasing content of Cl, namely 9.4 × 10^−5^ S/cm for NPSC-0, 1.4 × 10^−4^ S/cm for NPSC-1, 2.1 × 10^−4^ S/cm for NPSC-2, 4.2 × 10^−4^ S/cm for NPSC-3, 7.9 × 10^−4^ S/cm for NPSC-4, and 1.2 × 10^−3^ S/cm for NPSC-5. The ionic conductivity of the NPSC-5 sample (Na_5.5_PS_4.5_Cl_1.5_ with *x* = 0.5) showed the highest ionic conduction, which was over a hundred times higher than that of Na_6_PS_5_Cl (1.0 × 10^−5^ S/cm [40]), proving the importance of increasing the Cl content for Na^+^ migration. The NPSC-06 sample displayed a lower ionic conductivity value (2.9 × 10^−4^ S/cm) than that of NPSC-5 (Figure S4), which could be attributed to the low ionic conductivity of NaCl impurity (Figure S2). The whole testing and disassembly process of the steel/NPSC-5/steel configuration was completed in one shot, which can be observed in Video S1.

**Figure 5:**
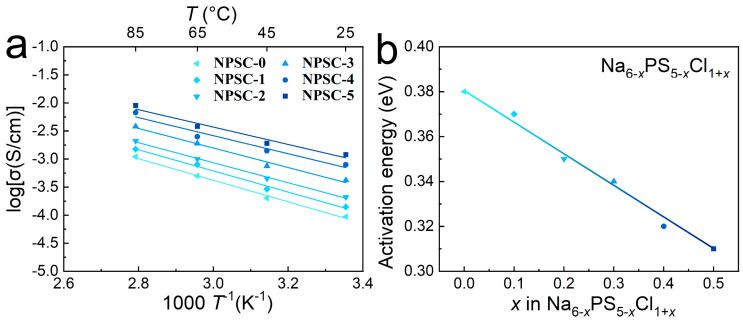
(**a**) Temperature dependence of the ionic conductivity of XRD patterns of Na_6−_*_x_*PS_5−_*_x_*Cl_1+*x*_ (*x* = 0, 0.1, 0.2, 0.3, 0.4, and 0.5, denoted as NPSC-0, NPSC-1, NPSC-2, NPSC-3, NPSC-4, and NPSC-5, respectively) samples. (**b**) Activation energies of Na_6−_*_x_*PS_5−_*_x_*Cl_1+*x*_ at different *x* values.

All the obtained samples exhibited linear relationships with ionic conductivity in the range of 25–85 °C. Figure 5a illustrates the temperature dependence of ionic conductivity. All obtained samples exhibited linear relationships between ionic conductivity and reciprocal temperature within the studied temperature range, indicating adherence to the Arrhenius law. By applying the Arrhenius equation (*σ*T = *σ*_0_exp(−*Ea*/*k_B_T*), where *σ*_0_ references a pre-exponential factor) [49], the activation energy (*E*_a_) of Na_6−_*_x_*PS_5−_*_x_*Cl_1+*x*_ (*x* = 0, 0.1, 0.2, 0.3, 0.4, and 0.5) for total conduction was determined from the slope of the plots. In Figure 5b, the composition dependence of the samples’ activation energies (*E*_a_) are depicted. AC impedance analysis results revealed that the ionic conductivity of all chlorine-rich Na_6−_*_x_*PS_5−_*_x_*Cl_1+*x*_ samples was higher than that of the primordial Na_6_PS_5_Cl (*x* = 0) sample, with ionic conductivity increasing from *x* = 0 to 0.5. These findings indicate that a rise in chlorine contents can enhance the ionic conductivity of Na_6_PS_5_Cl with a decrease in *E*_a_. Table S1 summarizes the ionic conductivities and *E*_a_ values reported in the literature, as well as the values obtained in this study. Compared with the reported Na_6_PS_5_Cl (0.01 × 10^−3^ S/cm) [40], the modified Na_5.5_PS_4.5_Cl_1.5_ exhibited superior ionic conductivity of 1.2 × 10^−3^ S/cm, which is the same order of magnitude as the Na_11_Sn_2_PS_12_ sulfide electrolyte (4 × 10^−3^ S/cm) reported in [35] and the vacancy-contained Na_3_SbS_4_ (3 × 10^−3^ S/cm) reported in [22]. Therefore, Na_5.5_PS_4.5_Cl_1.5_ (*x* = 0.5, NPSC-5) shows very high values of ionic conductivity among the typical sodium ion conductors that have been reported.

### 3.3. Electrochemical Performance of NVP//NPSC-5//Na ASSB

**Figure 6:**
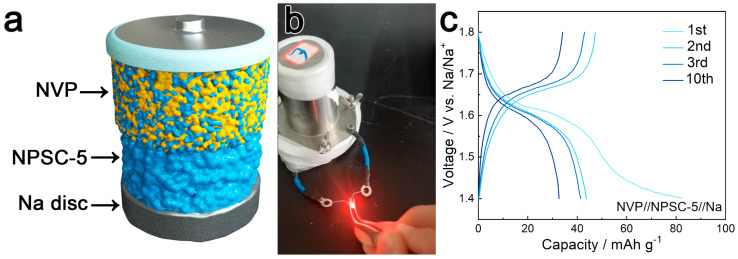
(**a**) Illustration of a Na_3_V_2_(PO_4_)_3_//NPSC-5//Na ASSB. (**b**) A digital picture of a Na_3_V_2_(PO_4_)_3_//NPSC-5//Na ASSB working at room temperature to make an LED diode bead light up. (**c**) Galvanostatic charge–discharge curves of a Na_3_V_2_(PO_4_)_3_//NPSC-5//Na ASSB at 25 °C with a charge–discharge speed of 0.05 C (1 C = 60 mA/g).

Na_5.5_PS_4.5_Cl_1.5_ (*x* = 0.5, NPSC-5) showed the highest ionic conductivity and lowest activation energy in the Na_6−*x*_PS_5−*x*_Cl_1+*x*_ system, so the electrochemical performance of a laboratory-grade, pressurized NVP//NPSC-5//Na cell with NPSC-5 as a sodium solid electrolyte and commercial Na_3_V_2_(PO_4_)_3_ (NVP, 99.9%, MTI Corporation, Shenzhen, China) and Na metal foil as cathode and anode, respectively, was determined, as depicted in the schematic diagram in Figure 5a. Since NVP can offer a 1.6 V voltage plateau through the V^3+^/V^2+^ redox reaction, which lies within the voltage stability region of the NPSC-5 electrolyte, the galvanostatic charge–discharge of the NVP//NPSC-5//Na ASSB was tested in the potential range of 1.4–1.8 V at a current density of 0.05 C. All charge–discharge profiles exhibited a 1.6 V plateau, corresponding to the phase transition of Na_3_V_2_(PO_4_)_3_/Na_4_V_2_(PO_4_)_3_ with one Na^+^ intercalation/deintercalation from the host lattices [50]. The right figure in Figure 6a shows a physical electronic photograph of an assembled all-solid-state sodium-ion battery that can normally light up an LED light bulb at room temperature, indicating that NPSC-5 is a pure sodium-ionic conductor material with negligible electronic conductivity and functions as a solid electrolyte. The additional plateau at about 1.42 V shown in Figure 6b can be attributed to the formation of a stable NPSC-5/Na interface during the first cycle, which was similar to reports of other electrochemical investigations sodium ASSBs [2,51,52,53]. The NPSC-5/Na interface resulted in a low coulombic efficiency (58%) during the first cycle (The initial discharge capacity was about 81.9 mAh/g, whereas the charge capacity was only 47.3 mAh/g). The second charge and discharge capacities were 43.1 mAh/g and 42.2 mAh/g, respectively, indicating the rebound of coulombic efficiency (98%). The 10th cycle displayed charge and discharge capacities of 34.3 and 33.7 mAh/g, respectively; nearly 73% of the initial charge capacity was retained, and the coulombic efficiency was maintained at 98%, demonstrating that the synthesized material facilitated the cyclic charging and discharging capabilities of the battery. The energy density of the full cell was about 70 Wh/Kg based on the mass of the active cathode. Further modifications, such as to the tips for battery assembly, compositing the NVP cathode with graphene, or replacing the Na disc with a Na-Sn alloy to improve the performance of the NVP//NPSC-5//Na ASSB, should be analyzed and explored in depth in the future.

## 4. Conclusions

In summary, halide-rich solid solutions of argyrodite-type Na_6−_*_x_*PS_5−_*_x_*Cl_1+*x*_ were synthesized and formed solid solutions in the range of 0 ≤ *x* ≤ 0.5. Increasing Cl^−^ contents effectively reduced the activation barrier and increased the Na-ion mobility, as well as electrochemical stability. The optimal solid solution composition of Na_5.5_PS_4.5_Cl_1.5_ exhibited particularly high Na-ion conductivity in the Na_6−_*_x_*PS_5−_*_x_*Cl_1+*x*_ in this work, namely 1.2 mS/cm at 25 °C, which is more than a hundred times higher than that of Na_6_PS_5_Cl with a regular composition. Meanwhile, Na_5.5_PS_4.5_Cl_1.5_ is more suitable for high-voltage cathodes than Na_6_PS_5_Cl due to its wider voltage stability region up to 2 V, and the smooth charge–discharge cycling performance of Na_3_V_2_(PO_4_)_3_//Na_5.5_PS_4.5_Cl_1.5_//Na at room temperature verifies that the obtained material is a pure sodium-ion conductor and its potential as a sodium-ionic solid electrolyte for all-solid-state sodium batteries. To date, the reported sulfide electrolyte, Na_11_Sn_2_PS_12_ (4 × 10^−3^ S/cm) [35] and vacancy-contained Na_3_SbS_4_ (3 × 10^−3^ S/cm) [22] have exhibited excellent conductivity. Both references proposed the strategy of designing Na vacancy for the improvement of conductivity. Therefore, we suppose that the ionic conductivity of Na_5.5_PS_4.5_Cl_1.5_ could be further promoted by designing Na vacancy in the crystal structure.

## Data Availability

Data are contained within the article.

## Supplementary Materials

The following supporting information can be downloaded at: https://www.mdpi.com/article/10.3390/ma17091980/s1, Figure S1: Comparison of XRD patterns for Na_6_PS_5_Cl, cubic Ag_15_P_4_S_16_Cl, tetragonal Na_3_PS_4_, cubic Na_3_PS_4_, and NaCl; Figure S2: XRD patterns of NPSC-5 and NPSC-6 samples and standard-NaCl; Figure S3: SEM images of (a) NPSC-0, (b) NPSC-1, (c) NPSC-2, (d) NPSC-3, and (e) NPSC-4 samples; Figure S4: The equivalent circuit used to extract the value of resistance from EIS spectra with two semicircles. All EIS spectra of Na_6−*x*_PS_5−*x*_Cl_1+*x*_, in general, exhibit two semicircular patterns. The conductivity was computed from the resistance using the following formula: Conductivity = Thickness/(Area × Resistance); Figure S5: Impedance plot of NPSC-6 sample at 25 °C; Table S1: Comparison of ionic conductivity and activation energy (*E*_a_) obtained in this study with those previously reported; Video S1: Impedance test of NPSC-5 sample.
