# Supplementary material for: Chlorine-Rich Na6−xPS5−xCl1+x: A Promising Sodium Solid Electrolyte for All-Solid-State Sodium Batteries
Source: Materials (Basel). 2024 Apr 24;17(9):1980. doi: 10.3390/ma17091980 (PMC11084612; doi:10.3390/ma17091980)
Supplement: Supplementary file 1 [file materials-17-01980-s001.zip › 3.Supporting Information.pdf]

Supporting Information

Figures and Tables

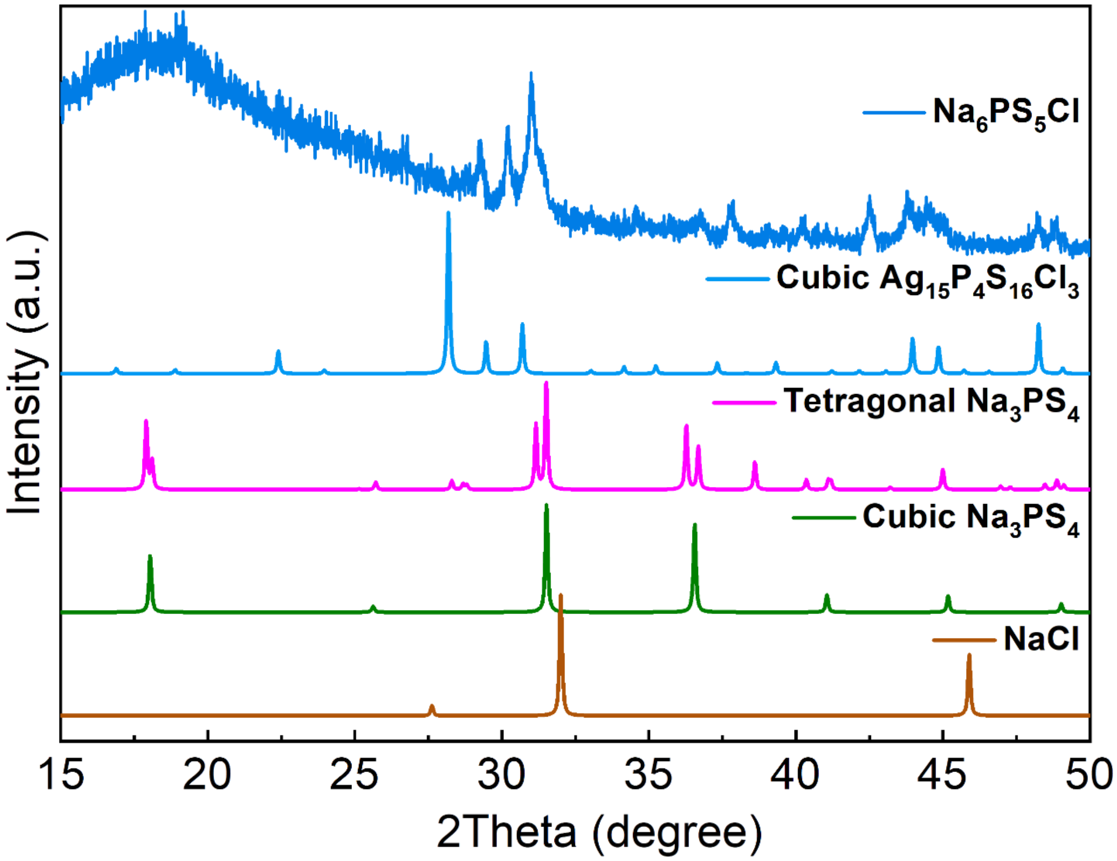

Figure S1. Comparison of XRD patterns for Na_6PS_5Cl, Cubic $Ag_{15}P_4S_{16}Cl$, Tetragonal Na_3PS_4, Cubic Na_3PS_4 and NaCl.

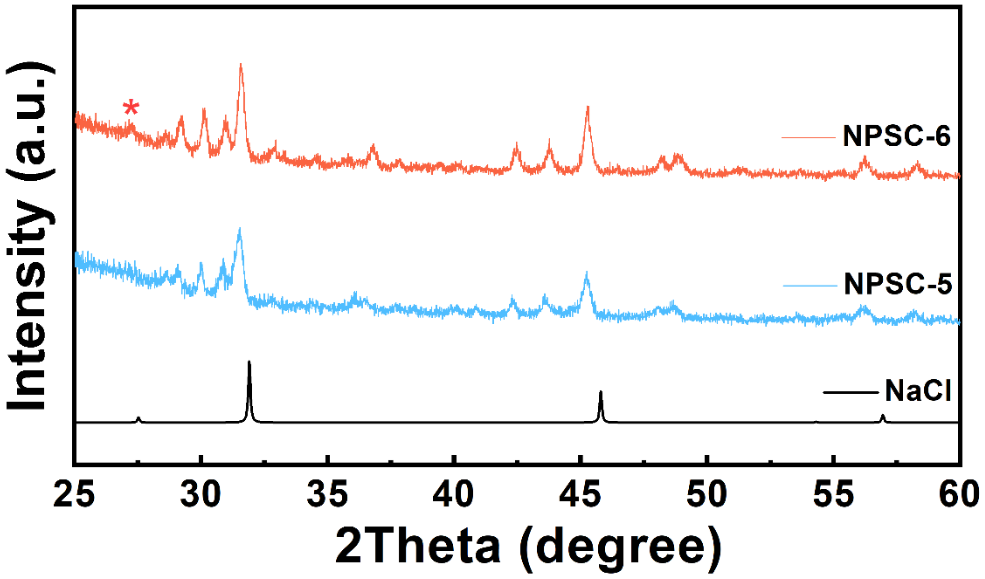

Figure S2. XRD patterns of NPSC-5, NPSC-6 samples and standard-NaCl.

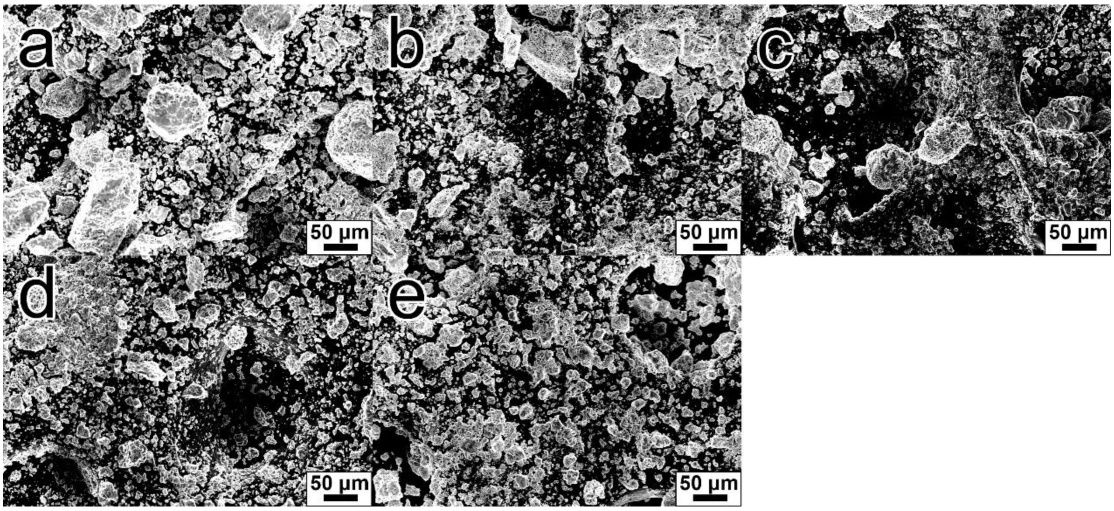

Figure S3. SEM images of (a) NPSC-0 (b) NPSC-1 (c) NPSC-2 (d) NPSC-3 (e) NPSC-4 samples.

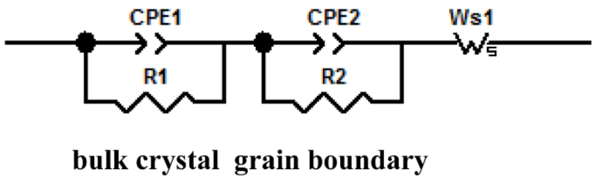

Figure S4. The equivalent circuit used to extract the value of resistance from EIS spectra with two semi-circles. All the EIS spectra of $Na_{6-x}PS_{5-x}Cl_{1+x}$ in general exhibit two semi-circles. The conductivity is computed from the resistance using the formula: Conductivity = Thickness/(Area×Resistance).

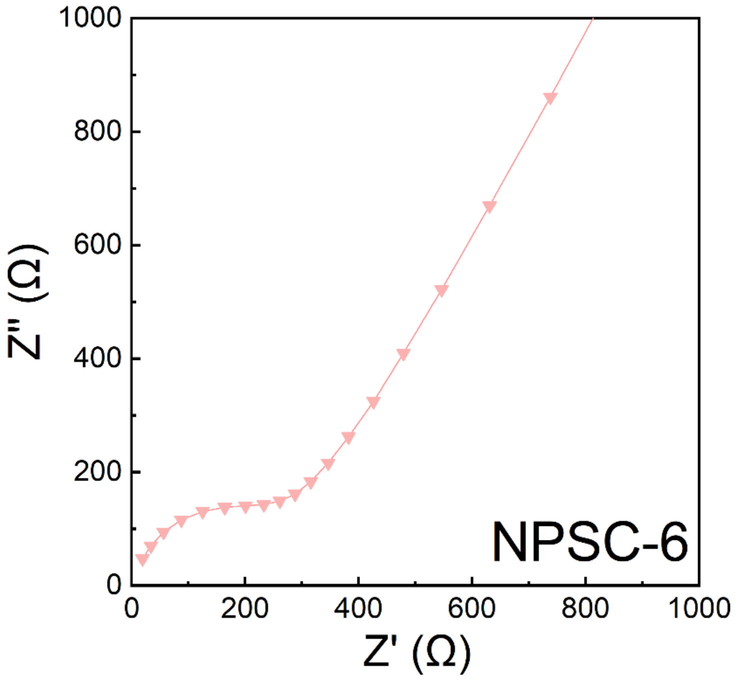

Figure S5. Impedance plot of NPSC-6 sample at 25 °C.

Table S1. The comparison of ionic conductivity and activation energy (E_a) in this study with those reported.

**Sulfide-type Sodium ionic conductors**	**Ionic conductivity (mS/cm)**	**E_a (eV)**	**ref**
$Na_{2.9375}PS_{3.9375}Cl_{0.0625}$	1.14	0.25	[1]
$Na_{2.730}Ca_{0.135}PS_4$	0.94	0.35	[2]
$Na_{10}SnP_2S_{12}$	0.40	0.36	[3]
$Na_{11}Sn_2PS_{12}$	1.40	0.25	[4]
$Na_{11}Sn_2PS_{12}$	4.00	0.38	[5]
Na_3SbS_4	3.00	0.25	[6]
Na_6PS_5Cl	0.01	none	[7]
**$Na_{5.5}PS_{4.5}Cl_{1.5}$**	1.20	0.31	This work

References

1. Chu, I.-H.; Kompella, C.S.; Nguyen, H.; Zhu, Z.; Hy, S.; Deng, Z.; Meng, Y.S.; Ong, S.P. Room-Temperature All-solid-state Rechargeable Sodium-ion Batteries with a Cl-doped Na_3PS_4 Superionic Conductor. *Sci. Rep.* **2016**, *6*, 33733.
2. Moon, C.K.; Lee, H.-J.; Park, K.H.; Kwak, H.; Heo, J.W.; Choi, K.; Yang, H.; Kim, M.-S.; Hong, S.-T.; Lee, J.H.; et al. Vacancy-Driven Na^+ Superionic Conduction in New Ca-Doped Na_3PS_4 for All-Solid-State Na-Ion Batteries. *ACS Energy Lett.* **2018**, *3*, 2504–2512.
3. Richards, W.D.; Tsujimura, T.; Miara, L.J.; Wang, Y.; Kim, J.C.; Ong, S.P.; Uechi, I.; Suzuki, N.; Ceder, G. Design and synthesis of the superionic conductor $Na_{10}SnP_2S_{12}$. *Nat. Commun.* **2016**, *7*, 11009.
4. Zhang, Z.; Ramos, E.; Lalère, F.; Assoud, A.; Kaup, K.; Hartman, P.; Nazar, L.F. $Na_{11}Sn_2PS_{12}$: A new solid state sodium superionic conductor. *Energy Environ. Sci.* **2018**, *11*, 87–93.
5. Duchardt, M.; Ruschewitz, U.; Adams, S.; Dehnen, S.; Roling, B. Vacancy-Controlled Na^+ Superion Conduction in $Na_{11}Sn_2PS_{12}$. *Angew. Chem. Int. Ed.* **2018**, *57*, 1351–1355.
6. Zhang, L.; Zhang, D.; Yang, K.; Yan, X.; Wang, L.; Mi, J.; Xu, B.; Li, Y. Vacancy-Contained Tetragonal Na_3SbS_4 Superionic Conductor. *Adv. Sci.* **2016**, *3*, 1600089.
7. Studenyak, I.P.; Pogodin, A.I.; Studenyak, V.I.; Kokhan, O.P.; Azhniuk, Y.M.; Solonenko, D.; Daróczi, L.; Kökényesi, S.; Zahn, D.R.T. Structural, electrical and optical properties of ion-conducting Na_6PS_5Cl, Na_6PS_5Br, and Na_7PS_6 compounds. *J. Phys. Chem. Solids* **2021**, *159*, 110269.
